# Ultralow thermal sensitivity of phase and propagation delay in hollow core optical fibres

**DOI:** 10.1038/srep15447

**Published:** 2015-10-22

**Authors:** Radan Slavík, Giuseppe Marra, Eric Numkam Fokoua, Naveen Baddela, Natalie V. Wheeler, Marco Petrovich, Francesco Poletti, David J. Richardson

**Affiliations:** 1 Optoelectronics Research Centre, University of Southampton, Southampton, SO17 1BJ, UK; 2 National Physical Laboratory, TW11 0LW Teddington, UK

## Abstract

Propagation time through an optical fibre changes with the environment, e.g., a change in temperature alters the fibre length and its refractive index. These changes have negligible impact in many key fibre applications, e.g., telecommunications, however, they can be detrimental in many others. Examples are fibre-based interferometry (e.g., for precise measurement and sensing) and fibre-based transfer and distribution of accurate time and frequency. Here we show through two independent experiments that hollow-core photonic bandgap fibres have a significantly smaller sensitivity to temperature variations than traditional solid-core fibres. The 18 times improvement observed, over 3 times larger than previously reported, makes them the most environmentally insensitive fibre technology available and a promising candidate for many next-generation fibre systems applications that are sensitive to drifts in optical phase or absolute propagation delay.

Optical fibres enable the propagation of optical signals over large distances. Although the intensity (power) of a propagating signal is relatively insensitive to fluctuations in the ambient temperature, its phase, ϕ, and propagation time, τ, through the fibre are not. A standard single-mode telecoms fibre suffers from a propagation time temperature sensitivity dτ/dT of 39 ps/km/K (at 1550 nm wavelength)^1^,^2^. This can pose significant challenges in many diverse application areas of optical fibres in physics and engineering. Primary examples lie in applications in which very precise timing signals need to be disseminated for synchronization purposes in large experimental infrastructures such as synchrotrons (e.g., FLASH in Desy, Hamburg^3^), linear particle accelerators, large telescope arrays, and in phase arrayed antennae. A value of 39 ps/km/K equates to a phase temperature sensitivity of about 48 rad/m/K. This can adversely affect many applications relying on fibre interferometers (e.g. fibre optic sensors^4^, quantum-optics^5^, interferometric measurement techniques, and so on), in which maintaining stable interference would require challenging temperature stabilization, often well below the sub-mK level. Similarly, a few key optical metrology applications require the dissemination of ultra-accurate optical signals over very long distances whilst maintaining propagation delay changes below 1 ps over many hours or days of operation. For example, the comparison of state-of-the-art optical clocks between National Measurement Institutes requires accuracy as high as parts in 10^−18^ to be preserved over hundreds or thousands of kilometers^6^. Such a level of precision is easily compromised by thermally-induced changes in optical path length (temperature drift) with time that unavoidably result in a Doppler frequency shift^6^.

The effect of such thermal sensitivity can, to a certain degree (and often to a currently-acceptable level), be mitigated by clever engineering solutions. Typical approaches include the use of a ‘common-path’ to cancel thermal drift (e.g. the two polarization modes of the same fibre are used to define the two arms of an interferometer^7^, two propagation directions within a Sagnac loop interferometer^4^, etc.). Another example is active stabilization of the optical path length exploiting propagation of the same signal in the same fibre bi-directionally, which may be impaired due to processes such as Rayleigh scattering that can cause interference between forward and backward propagating signals. This is detrimental especially when optical amplification is needed (e.g., using an erbium-doped fibre amplifier), as the back-scattered signal provides unwanted feedback into the amplifiers.

Optical fibres with a much reduced thermal sensitivity would provide a simpler (and thus more robust and more reliable) ‘passive’ solution for many of these applications. Indeed, this new type of fibre might even open new application areas where current technology is still too temperature sensitive to be used (e.g., as a reference resonator^8^).

To this end, specialty coatings have been developed that can be applied to a solid SMF-28 fibre to counteract thermally induced fibre elongation and refractive index changes^1^. The very best coated fibres are able to reduce dτ/dT down to 3.7 ps/km/K^1^, representing up to an order of magnitude reduction relative to standard SMF-28 fibre. Such fibres are now commercially available (e.g. the ‘Phased-Stabilized Optical Fiber Cable’ from Furukawa Japan and the STFOC Optical Cable from Linden Photonics, U.S.A). Although this level of reduction is useful in many applications, further reduction in temperature sensitivity is still highly desirable. Moreover, (Johnson) white thermal noise (detrimental in fibre lasers for example^9^), is unlikely to be suppressed by a special coating, especially at high frequencies, due to the limited thermal diffusivity (heat propagation speed across the fibre cross section).

It is well known that the thermal sensitivity of fibre dτ/dT has two main contributions^2^: the thermal dependence of the glass refractive index and the change of the fibre length due to temperature variations. Experiments have shown that in solid silica fibres the first term is about 20 times larger than the second one^2^ (and thus contributes 95% of the total thermal sensitivity), suggesting that a change in the light guiding material (fibre core) may reduce the thermal sensitivity significantly. Consequently, Hollow-Core Photonic Bandgap Fibres (HC-PBGFs), in which light is predominantly guided in an air core with negligible thermal refractive index coefficient should be expected to have around 20 times lower dτ/dT as compared to solid-core silica fibres. However, a previous study of the thermal sensitivity of HC-PBGFs only reported an improvement by a factor of between 3 and 5 as compared to SMF-28^4^, which is significantly worse than is expected from the considerations above.

In order to address this inconsistency, here we revisit the issue of thermal sensitivity of HC-PBGF, through two different and independently executed experiments. We show that HC-PBGFs actually have over 18 times lower thermal sensitivity as compared to SMF-28, which is almost as good as expected (from the considerations above). This removes the discrepancy between the expectations and the experimental observation, which forms the main scientific aspect of our paper. Further, we show that HC-PBGFs are the best-performing fibres available today in terms of thermal sensitivity, showing about two times lower sensitivity than the best-performing published specialty-coated solid-core fibres, making our findings important for relevant applications that span—as previously outlined—several research fields.

Let us first present a simple analysis to estimate the thermal sensitivity of solid and hollow core fibres, which can be understood through a basic examination of the contributions to the dτ/dT of the fibre. First, the transit time for signal propagation through an optical fibre is:


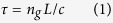


where *L* is the fibre length, *n*_*g*_ the group index of the mode and *c* is the speed of light in vacuum. The change in transit time with temperature is thus expressed as:


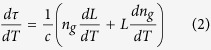


The first term quantifies the effect of fibre elongation, while the second term quantifies the effect of variations in *n*_*g*_ through thermo-optic and elasto-optic effects. For fused silica (at 1550 nm wavelength)^10^: 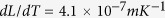 (for *L* *=* *1* *m*) and 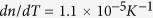. Therefore, if we omit the small variations due to glass dopants in the core and approximate *n*_*g*_ with the glass refractive index *n*, we find that the 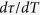 contributions are:


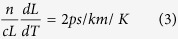


for the temperature induced length variation and


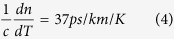


for the temperature induced refractive index variation. In line with explanations presented in ref. 1, we neglect the contribution of the polymer fibre jacket (due to the weak mechanical coupling between the fibre glass and the jacket). The resulting value of 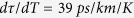 is close to the values observed experimentally for a single-mode fibre^1^. In a HC-PBGF most of the light (typically >99%^11^) propagates in air which has a negligible thermo-optic coefficient, thus we expect *dτ/dT* to be determined predominantly by the fibre elongation (responsible for 2 ps/km/K). Indeed we would expect the contribution from fibre elongation to be even lower in HC-PBGF since *n*_*g*_ is about 1.003 as compared to 1.45 in silica-core fibre. Thus, based on Equation (3) we would expect a value of 1.42 ps/km/K. Since for our HC-PBGF only about 0.3% of the guided light propagates in the glass, we can estimate the contribution from the refractive index variation using Equation (4) to be ~0.13 ps/km/K. This gives us a total estimate for HC-PBGFs of 1.6 ps/km/K.

## Results

**Figure 1:**
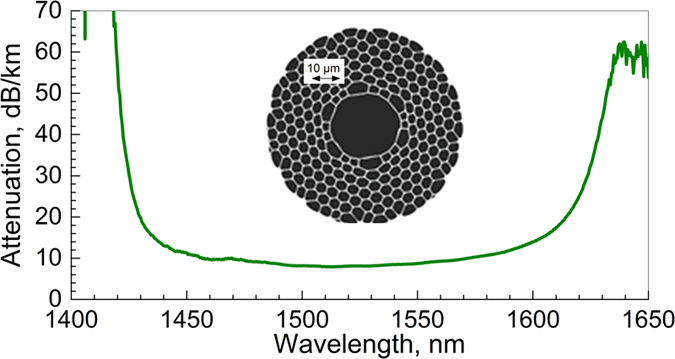
Spectral attenuation of the HC-PBGF used. The inset shows the SEM image of the fibre microstructure.

For our thermal sensitivity measurements we used a 19-cell HC-PBGF similar to the one we previously used to demonstrate low latency data transmission^11^. The fibre outer diameter was 165 μm and the core diameter was 29.2 μm. Mode field diameter (MFD) was calculated to be ~21.5 μm at 1550 nm wavelength and air filling fraction was estimated to be ~90%. Calculated group velocity dispersion of the fundamental guided mode at 1550 nm wavelength was ~27 ps/nm/km. The transmission loss of this fibre is shown in Fig. 1 together with a Scanning Electron Micrograph (SEM) image showing the microstructure of the core. It is worth mentioning that the fibre has a larger core than those used in ref. 4, which helps to decrease the fractional power in the glass from an estimated 1%^4^ to about 0.3%, which is a value obtained from simulations considering the fiber parameters and geometry extracted from the SEM image (inset of Fig. 1).

**Figure 2:**
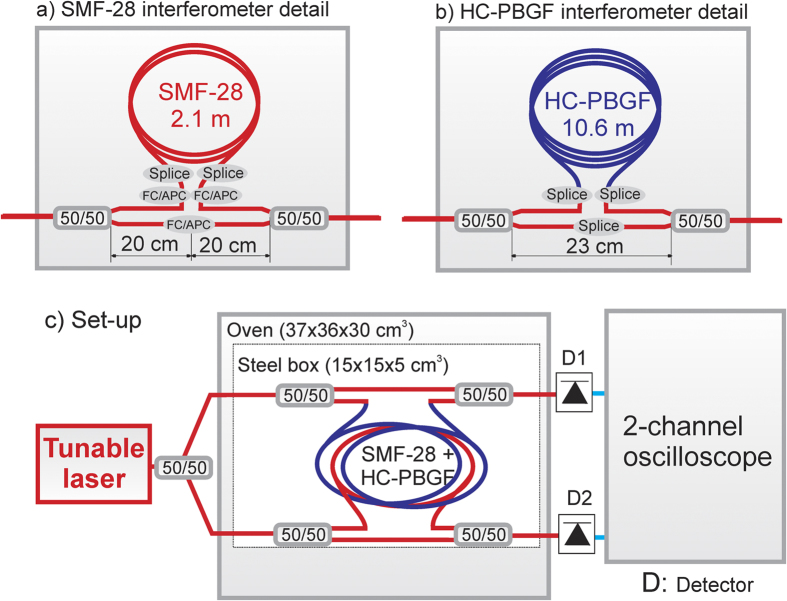
Measurement arrangement for the first test. Details of the two interferometers (including pigtail lengths that were kept as short as possible, FC/APC connector points, and splices points) are shown in (**a**,**b**). HC-PBGF is shown in blue while SMF-28 is shown in red. Both interferometers are placed together in a steel box. The two fibres (SMF and HC-PBGF) are wound closely together (**c**).

We carried out two independent tests. In the first test, undertaken at the University of Southampton, two fibres—a 10.6-m long HC-PBGF and a 2.1-m long Corning SMF-28e+ were wound together in loops with a diameter of 9 cm and fixed to a sheet of cardboard. The length of SMF-28 was chosen to be shorter, since its thermal sensitivity was expected to be significantly larger than that of the HC-PBGF. These two samples were incorporated into two distinct Mach-Zehnder fibre interferometers—details are shown in Fig. 2. The two interferometers were put together into a small metallic box (15 × 15 × 5 cm^3^), which was then placed into an oven. Further details are provided in the Methods section and in Fig. 2.

**Figure 3:**
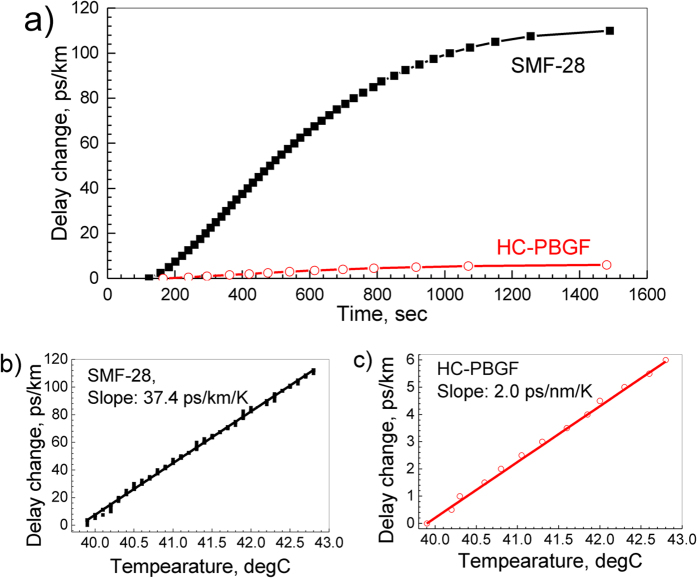
Normalized delay change in the two interferometers. The delay is shown as a function of time after the oven temperature setting was changed from 40 to 43 °C (**a**). The dependence on temperature is shown in (**b**,**c**) together with a linear fit that gives the thermal sensitivity of 37.4 ps/km/K (SMF-28, (**b**)) and 2.0 ps/km/K (HC-PBGF, (**c**)). Results measured at λ = 1535 nm.

**Figure 4:**
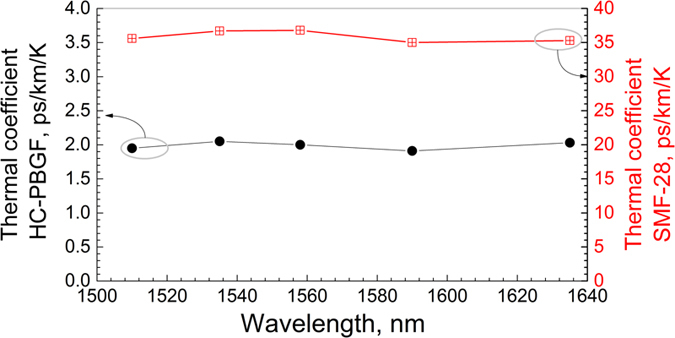
Thermal coefficient for HC-PBGF and SMF-28 as a function of wavelength.

The propagation time difference (delay) between the two arms of the imbalanced interferometer when the trace recorded on the oscilloscope goes from a maximum to a minimum is


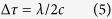


where λ is the wavelength of the light used in vacuum. From this, the total variation in delay as a function of time (hence temperature) can be reconstructed. Figure 3a shows the result of a measurement at λ = 1535 nm, where the time delay has been re-normalized to 1 km of fibre. As can be seen, it took about 1500 s for our oven to stabilize to the new temperature. The dependence of the delay on temperature is shown in Fig. 3b,c. As expected, this dependence is linear, corresponding to dτ/dT = 37.4 ps/km/K for SMF-28 and 2.0 ps/km/K for our HC-PBGF. The value obtained for SMF-28, dominated by refractive index variation, is in good agreement with previously-reported values^1^ and with Equation (4), despite the fact that the effect of glass dopants or changes in waveguide properties due to the temperature are neglected in Equation (4). Similarly, for HC-PBGF, the value is reasonably close to the estimated value of 1.6 ps/km/K. We carried out the characterization at various wavelengths between 1510 and 1635 nm (the full tuning range of our tunable laser source). The resulting thermal coefficient for both fibres is shown in Fig. 4 and it indicates the same behavior (within measurement error) across the entire spectral range. It is worth mentioning that the longest-wavelength measured (1635 nm) is very close to the bandgap edge (Fig. 1), where the *n*_*g*_ is expected to change relatively quickly (due to the Kramers-Krönig relation). However, we do not see any change in dτ/dT, confirming that the contribution of *n*_*g*_ to *dτ/dT* (Equation (4)) is significantly smaller than the contribution due to the fibre elongation (Equation (3)).

**Figure 5:**
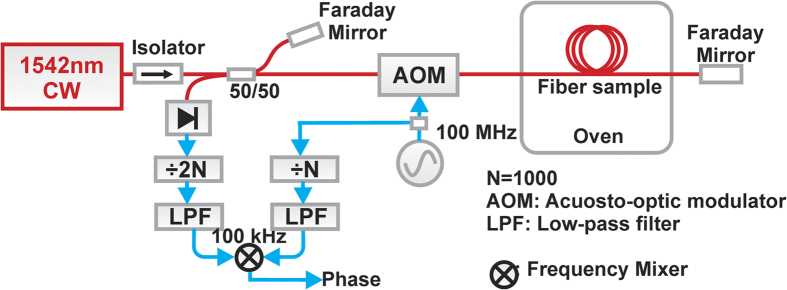
The second measurement set-up.

In the second experiment, performed independently at the UK National Physical Laboratory, different samples of HC-PBGF and SMF-28+ fibres (however, coming from the same batches of fibre used in the experiments at the University of Southampton) were used. The fibre samples were 20 m long (both SMF28e+ and HC-PBGF) and 1.5-m long pigtails made of SMF-28+ were spliced at each end. Each fibre sample was coiled separately, with a diameter of about 21 cm, and both were fixed onto a cardboard sheet. Most of the length of the SMF-28 pigtails was kept outside of the oven, leaving just 5–6 cm inside. This length was covered in insulating foam. The air temperature inside the oven was cycled by switching the oven on and off every 1000 seconds, leading to a peak-to-peak temperature change of 0.5 K. In contrast with the previous experiment, the sensitivity characterization was performed with only one fibre sample at a time inside the oven. The measurement set-up used is shown in Fig. 5; the details regarding experimental set-up are provided in the Methods section.

**Figure 6:**
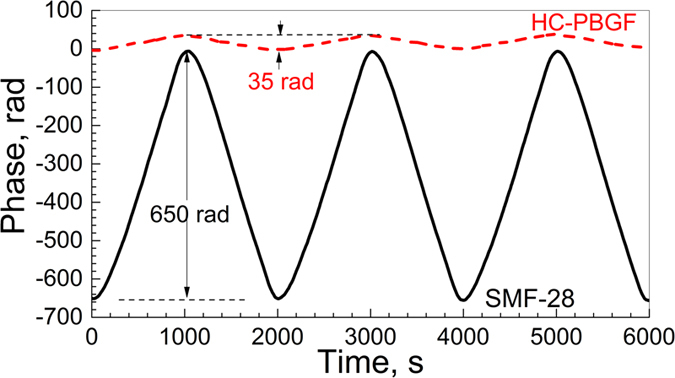
Phase change of the light measured with the 2^nd^ set-up. The light propagated through 20-m lengths of SMF-28 (solid) and HC-PBGF (dashed). The air temperature in the oven was changed by 0.5 K peak-to-peak every 1000 s.

The results are shown in Fig. 6. Here, we see that the SMF-28 exhibited a peak-to-peak phase deviation of 650 rad, while for HC-PBGF, the measured phase deviation was only 35 rad, confirming the 18.5 times sensitivity reduction observed in the first experiment.

We also measured the sign of the temperature change-induced Doppler shift and found it to be the same for both fibres. This means that the sign of the time delay temperature sensitivity coefficient dτ/dT is the same for both fibres, as we expected from the theory.

## Discussion

We have carried out two independent measurements to measure the thermal sensitivity of signal propagation time through a HC-PBGF and compared it with that of SMF-28. HC-PBGF was found to have a sensitivity of 2 ps/km/K, around 18.5 times smaller than that of SMF-28.

Our results show that HC-PBGFs offer a significantly better performance than calculated and measured previously^4^. While it is hard to speculate as to the reasons for the experimental discrepancy with the previously-published measurement, only a small part of which can be explained by the use of a fibre with lower overlap between the guided mode and the fibre glass, the agreement shown here between two different experiments leave us confident about the reliability of our conclusion. In terms of the discrepancy with the earlier theory, we believe that this originates from the assumption in ref. 4 that the fibre jacket would play a significant role, which later experimental studies have proved incorrect^1^.

Most importantly, our-measured value of thermal sensitivity for a HC-PBGF with a standard acrylate coating is almost a factor of 2 better than the best-performing low-sensitivity fibre available on the market (based on SMF-28 and a tight specialty coating with opposite thermal expansion coefficient). The specialty fibre coatings used on the SMF-28 low-sensitivity fibres have a more limited operational temperature range (e.g., up to about 100 °C), while a standard coated/uncoated HC-PBGF can maintain its properties up to significantly higher temperatures. For operation below 100 °C we expect that a suitable coating with opposite thermal expansion coefficient could also be used on the HC-PBGF, which could lead to a further reduction of its already-low sensitivity, e.g., by an order of magnitude, which was the level of improvement achieved with SMF-28. Additionally, we believe that the fibre design could be altered (different microstructure parameters) to further reduce the thermal sensitivity, as the bandgap wave-guiding properties (completely neglected in our simplistic analysis presented earlier) may be used to counter-balance the contribution from the fibre elongation. A change in the glass refractive index results in a small shift of the photonic bandgap that in turn can change the fraction of power carried in the core (or glass). This change can be positive or negative, depending on the wavelength of operation within the bandgap^12^, possibly providing means for compensation of the fibre elongation. As our experimental results (Fig. 4) show that this effect is negligible in our fibre, we are currently working on fibre designs to optimize this effect and thus possibly further reduce the HC-PBGF thermal sensitivity.

It is also to be appreciated that HC-PBGFs offer the low propagation time thermal sensitivity in conjunction with other unique characteristics. These primarily originate from the fact that most of the energy propagates through the air core rather than the fused silica material and include: ultra-low nonlinearity (of interest, for example, for single-mode fibre propagation of high-peak power pulses^13^); low latency data transmission^11^; and high radiation hardness^14^. The combination of these unique properties makes this fibre attractive for a range of applications including gyroscopes, fibre interferometers and delivery of precise synchronization signals.

## Methods

### HC-PBGF splicing

In our experiments, the HC-PBGF was spliced directly at both ends to SMF-28 fibre using a standard telecom arc fusion splicer. Although this resulted in a relatively high loss (~5 dB/splice) this was adequate for the purpose of our measurements. The insertion loss could be reduced through the use of more sophisticated splicing procedures^15^. All the fibre splices were left unprotected to avoid any unwanted effects from thermal expansion of the splice protectors, as we observed such effects in preliminary tests in which splice protectors were used. This is because splice protectors are usually reinforced by a steel wire. However, steel has more than an order of magnitude larger thermal expansion coefficient to that of silica. Thus, although the splice protector is relatively short (20–40 mm), its impact on fibre elongation/stress can be significant, especially when measuring HC-PBGFs that have a very low thermal sensitivity coefficient.

### First measurement set-up (Fig. 2)

The interferometers’ reference arms contained short pigtails made of SMF-28 accurately matched (to within 1 cm) to the SMF-28 pigtails in the other arm. The pigtails were kept as short as possible and were taped together to ensure they experienced the same temperature. A signal from a narrow linewidth tunable laser (Agilent 81600B, option 160) was split via a 50/50 coupler and launched into both interferometers. Two photodetectors were used to simultaneously monitor the output of both interferometers as a function of time via a 2-channel oscilloscope, while the temperature was increased. The oven temperature was stabilized to 40 °C (well above the ambient temperature). After a suitable stabilization period (about 1 hour, when no drift in the interference pattern during 2 minutes was observed) we increased the oven temperature setting to 43 °C. The times at which the oscilloscope traces went through maxima and minima were manually recorded. At the same time, we also recorded a trace of the evolution of the temperature inside the box with time using a thermometer (resolution of 0.1 °C) which was attached to the fibres under test. No attempt was made to maximize the visibility of the interferometer fringes by incorporation of polarization control elements since we only needed to record the time of maximum/minimum interference to extract the temperature sensitivity.

### Second measurement set-up (Fig. 5)

The light from a CW narrow linewidth laser emitting at 1542 nm was split by a 50:50 splitter and sent through a ‘reference’ and a ‘measurement’ arm of a modified Michelson interferometer. A Faraday mirror on one of the two outputs of the 50:50 splitter was used to return the signal to a photodetector (reference arm). The frequency of the carrier at the other output of the 50:50 splitter (measurement arm) was shifted by 100 MHz using an acousto-optic modulator (AOM) and then propagated through the fibre. The optical carrier was then returned back into the fibre and the AOM using a fibre-coupled Faraday mirror. The resulting 200 MHz beat between the reference and measurement arm of the Michelson interferometer was detected and divided by a factor of 2000 using a digital divider, which resulted in a signal modulated at 100 kHz (2 × 100 MHz/2000). The reference signal was derived from the AOM drive signal (100 MHz tone) divided by 1000 using a second digital divider, resulting in a signal at 100 kHz. The frequency division was required in order to reduce the optical phase fluctuations induced by the fibre to lie within the linear range of the RF mixer used as the phase detector.

## Additional Information

**How to cite this article**: Slavík, R. *et al.* Ultralow thermal sensitivity of phase and propagation delay in hollow core optical fibres. *Sci. Rep.* **5**, 15447; doi: 10.1038/srep15447 (2015).
